# Intranasal powder live attenuated influenza vaccine is thermostable, immunogenic, and protective against homologous challenge in ferrets

**DOI:** 10.1038/s41541-021-00320-9

**Published:** 2021-04-21

**Authors:** Jasmina M. Luczo, Tatiana Bousse, Scott K. Johnson, Cheryl A. Jones, Nicholas Pearce, Carlie A. Neiswanger, Min-Xuan Wang, Erin A. Miller, Nikolai Petrovsky, David E. Wentworth, Victor Bronshtein, Mark Papania, Stephen M. Tompkins

**Affiliations:** 1 Center for Vaccines and Immunology, University of Georgia, Athens, Georgia USA; 2 Emory-UGA Centers of Excellence for Influenza Research and Surveillance (CEIRS), Athens, Georgia 30602 USA; 3 Influenza Division, Centers for Disease Control and Prevention, Atlanta, Georgia USA; 4 Universal Stabilization Technologies, Inc., San Diego, California USA; 5 College of Medicine and Public Health, Flinders University, Adelaide, South Australia Australia; 6 Vaxine Pty Ltd, Warradale, South Australia Australia; 7 Global Immunization Division, Centers for Disease Control and Prevention, Atlanta, Georgia USA; 8 Department of Infectious Diseases, University of Georgia, Athens, Georgia USA

**Keywords:** Preclinical research, Influenza virus, Vaccines, Live attenuated vaccines

## Abstract

Influenza viruses cause annual seasonal epidemics and sporadic pandemics; vaccination is the most effective countermeasure. Intranasal live attenuated influenza vaccines (LAIVs) are needle-free, mimic the natural route of infection, and elicit robust immunity. However, some LAIVs require reconstitution and cold-chain requirements restrict storage and distribution of all influenza vaccines. We generated a dry-powder, thermostable LAIV (T-LAIV) using Preservation by Vaporization technology and assessed the stability, immunogenicity, and efficacy of T-LAIV alone or combined with delta inulin adjuvant (Advax™) in ferrets. Stability assays demonstrated minimal loss of T-LAIV titer when stored at 25 °C for 1 year. Vaccination of ferrets with T-LAIV alone or with delta inulin adjuvant elicited mucosal antibody and robust serum HI responses in ferrets, and was protective against homologous challenge. These results suggest that the Preservation by Vaporization-generated dry-powder vaccines could be distributed without refrigeration and administered without reconstitution or injection. Given these significant advantages for vaccine distribution and delivery, further research is warranted.

## Introduction

Influenza viruses pose a dual threat to global human health—annual seasonal epidemics and sporadic global pandemics. Influenza vaccination is the best measure to counter both seasonal and pandemic influenza threats. Seasonal influenza epidemics, caused by antigenic drift, result in 3–5 million cases of severe illness and 290,000–650,000 deaths annually^1^. Annual vaccination is the most effective strategy for prevention and control of seasonal influenza viruses^2^. However, current influenza vaccines have characteristics that limit rapid, widespread delivery. Influenza vaccination strategies include parenteral injection of inactivated influenza vaccines (IIVs) and intranasal spray delivery of live attenuated influenza vaccines (LAIVs)^3^. LAIVs are based on genetically stable master donor viruses (MDVs), which express the surface glycoproteins of currently circulating viruses, and the internal proteins of the MDV^4,5^. Two influenza A MDVs have been generated: A/Ann Arbor/6/1960 (H2N2)^6^ and A/Leningrad/134/17/1957 (H2N2)^7^. Mutations in the internal protein coding gene segments confer attenuated (*att*), cold-adapted (*ca*), and temperature-sensitive (*ts*) phenotypes to these MDVs^4,5^, leading to replication that is limited to the upper respiratory tract. LAIV was first licensed for use in the United States in 2003^8^ and was initially released as a frozen formulation, although storage difficulties drove the development of a refrigerator-stable formulation^9^. LAIV4 is an intranasally administered, quadrivalent liquid LAIV containing the surface glycoproteins of two influenza A (H1N1 and H3N2) and two influenza B strains^10^. Intranasal vaccination with LAIV advantageously mimics the natural route of infection, eliciting robust mucosal immunoglobulin^11^ and cellular immune responses^12^. Although some LAIVs require healthcare workers (HCWs) to mix the dry vaccine with a diluent, intranasal delivery generally overcomes the challenges associated with parenteral injection with needle and syringe, such as the need for skilled HCW, which are in short supply in many settings, patient needle phobia, and biohazardous sharps waste.

Although LAIV provides some benefits over IIV, all current influenza vaccines require refrigeration from the time of manufacture to the time of delivery. Vaccine potency can be negatively affected if not maintained at adequately cold temperatures or if it is improperly exposed to freezing temperatures during storage. Indeed, exposure of LAIV to elevated temperatures during shipment coupled with reduced thermal stability of the H1 component was associated with reduced efficacy of liquid LAIV^13,14^. The requirement to maintain a reliable cold chain is a major logistical hurdle to vaccine distribution and delivery, and contributes to increased manufacturing, transportation, and storage costs. Improper vaccine storage leads to substantial product wastage^15^. Moreover, improper storage of vaccines results in the need to revaccinate, which may lead to loss of patient confidence^15^. Thermostable vaccines would ameliorate the loss of vaccine potency that results when the cold chain is interrupted, facilitate distribution in pandemic responses and mass vaccination campaigns, and ease the standard annual vaccination logistics. Building global capacity to respond to annual influenza epidemics, including developing vaccines that can be distributed without refrigeration and administered without injection, is one of the best ways to prepare for the next influenza pandemic.

The continued threat of the emergence of a novel pandemic strain of influenza A virus remains a major public health concern. A severe influenza pandemic could be similar in scale to the current coronavirus disease 2019 (COVID-19) pandemic, a devastating global health event with far reaching consequences. Recently, 2018 marked the centennial of the 1918 Spanish influenza (H1N1) pandemic, which claimed an estimated 50 million lives worldwide^16^. Subsequent influenza pandemics included the 1957 Asian (H2N2), 1968 Hong Kong (H3N2), and 2009 swine flu (H1N1) pandemics^17,18^. To mitigate this threat, pandemic preparedness frameworks have been developed by government and non-government entities, including the World Health Organization and the US government^19–21^. Key priorities of these pandemic preparedness plans include the capacity for rapid deployment of vaccines and to develop new vaccines as needed to respond to disease threats. Pandemic-ready vaccines should have characteristics such as single-dose regimens, reduced manufacturing cost, and increased thermostability—as storage requirements of temperature-sensitive vaccines limits capacity for rapid distribution in the event of a pandemic^22,23^. Furthermore, the National Institute of Allergy and Infectious Diseases has prioritized development of a universal influenza vaccine, highlighting key research objectives, including evaluation of alternate modes of delivery for currently available vaccines^24^.

To circumvent the need for the cold chain, dry-powdered vaccines that are stable at ambient temperature have been formulated against many bacterial and viral pathogens of human concern^25^, although none are currently commercially available. Dry-powder vaccine formulations are commonly generated by freeze-drying (lyophilization) or spray drying^26^; however, these processes can be detrimental to biologics such as vaccines^26,27^. Changes in buffer composition, pH, or the formation of ice crystals may lead to altered protein conformation or mechanical damage to the immunizing antigen, potentially reducing the potency of the vaccine^26,27^. To mitigate the adverse effects of freeze or spray drying, Preservation by Vaporization (PBV) technology preserves vaccines and other biologics in a glassy state by primary drying of the vaccine, followed by a stability drying stage. Primary drying involves intensive vaporization of water from biological products (sublimation, boiling, and vaporization), leading to mechanical stability of the vaccine formulation at ambient temperature, while under high vacuum. Stability drying increases the glass transition temperature of the vaccine to sustain the mechanical stability at ambient temperatures outside of vacuum^28^. Immobilization of vaccines in glassy matrices significantly reduces diffusion and molecular mobility, minimizing vaccine degradation and imparting thermostable properties^26,29^. PBV-generated vaccines can be reconstituted with water in the vial for delivery by injection or liquid intranasal spray or can be micronized into a powder for direct mucosal delivery in needle-free formats. PBV is efficient, economical, and is suitable for commercial production lines to generate mass quantities of dry-powder, thermostable influenza vaccines for intranasal delivery.

Immunogenicity and effectiveness of LAIV vaccines remains an issue in some populations, particularly in older individuals, which is generally ascribed to pre-existing antibody immunity that reduces infectivity and thereby efficacy of the LAIV in this population^30–32^. Although adjuvants have traditionally been used to increase the immunogenicity of inactivated vaccines, there is a paucity of data on use of adjuvants to enhance effectiveness of live attenuated vaccines^33–35^. Advax™ is a novel polysaccharide adjuvant derived from the plant sugar, delta inulin, which was developed through the National Institutes of Health’s Adjuvant Development Program^36,37^. Its dry-powder format and rapid reconstitution makes it well-suited for mucosal administration. Advax™ has been shown to enhance vaccine immunogenicity and protection across a broad range of vaccines including parenterally administered inactivated and recombinant seasonal and pandemic influenza vaccines^38–44^, with confirmation of its efficacy and safety in multiple human clinical trials^45–47^. We studied whether the addition of Advax™ enhanced thermostable LAIV (T-LAIV) effectiveness.

In this study, LAIV (T-LAIV) created with the A/Leningrad/134/17/1957 MDV was thermostabilized at ambient temperatures using PBV (US Patent No. 9,469,835^28^). Subsequently, ball milling [Laarmann Lab Wizz ball mill] was used to micronize the dry-powder formulation for intranasal delivery and the stability the T-LAIV stored at ambient temperature (25 °C) for up to 1 year was determined using infectivity assays. Immunogenicity of T-LAIV with and without Advax™ adjuvant was examined using the ferret influenza model and protective efficacy against homologous challenge was assessed. The results showed that PBV-generated thermostable T-LAIV retained infectivity when stored at ambient temperature (25 °C) for at least 1 year, was compatible with Advax™ adjuvant, and induced mucosal IgG and robust serum hemagglutination inhibition (HI) responses to protect ferrets challenged with an antigenically matched influenza strain. This work demonstrates that PBV is a potential platform process for manufacturing thermostable dry-powder influenza vaccine candidates for intranasal delivery.

## Results

### PBV-thermostabilized LAIV maintains infectivity during high-temperature storage

**Fig. 1:**
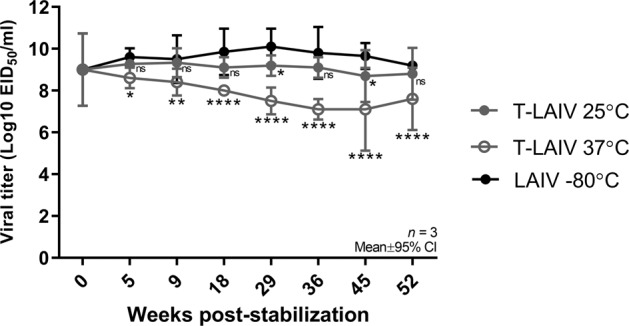
Infectivity of PBV-stabilized live attenuated influenza vaccine. Thermostable LAIV was stored at 25 °C and 37 °C, and liquid LAIV was stored at −80 °C for up to 52 weeks. T-LAIV was reconstituted with 1 ml PBS and infectious titer was determined by titration in embryonated chicken eggs. T-LAIV stability at each time point was compared to liquid LAIV stored at −80 °C (black circles). Two-way ANOVA: *p < 0.05, **p < 0.01, ****p = 0.0001; ns, not significant. Error bars indicate mean ± 95% CI. *n* = 3.

To assess the stability of PBV-stabilized T-LAIV, aliquots containing 1 × 10^9^ EID_50_/ml T-LAIV were incubated at 25 °C or 37 °C for up to 1 year and infectious virus titer was determined by titration in embryonated chicken eggs (ECEs). T-LAIV stability at each time point was compared to liquid LAIV stored at −80 °C. T-LAIV was highly stable when stored at an ambient temperature; there was no significant loss in titer of T-LAIV stored at 25 °C for 52 weeks (10^8.8^ EID_50_/ml) when compared to liquid LAIV controls stored at −80 °C for 52 weeks (10^9.2^ EID_50_/ml) (Fig. 1). T-LAIV stored at an elevated temperature resulted in a modest loss of infectivity over time. T-LAIV stored at 37 °C lost one log_10_ of infectious titer by week 18 (10^8.0^ EID_50_/ml) and a 1.5 log_10_ reduction at week 52 (10^7.6^ EID_50_/ml).

### Dry-powder T-LAIV efficiently replicates in the upper respiratory tract of ferrets

**Fig. 2:**
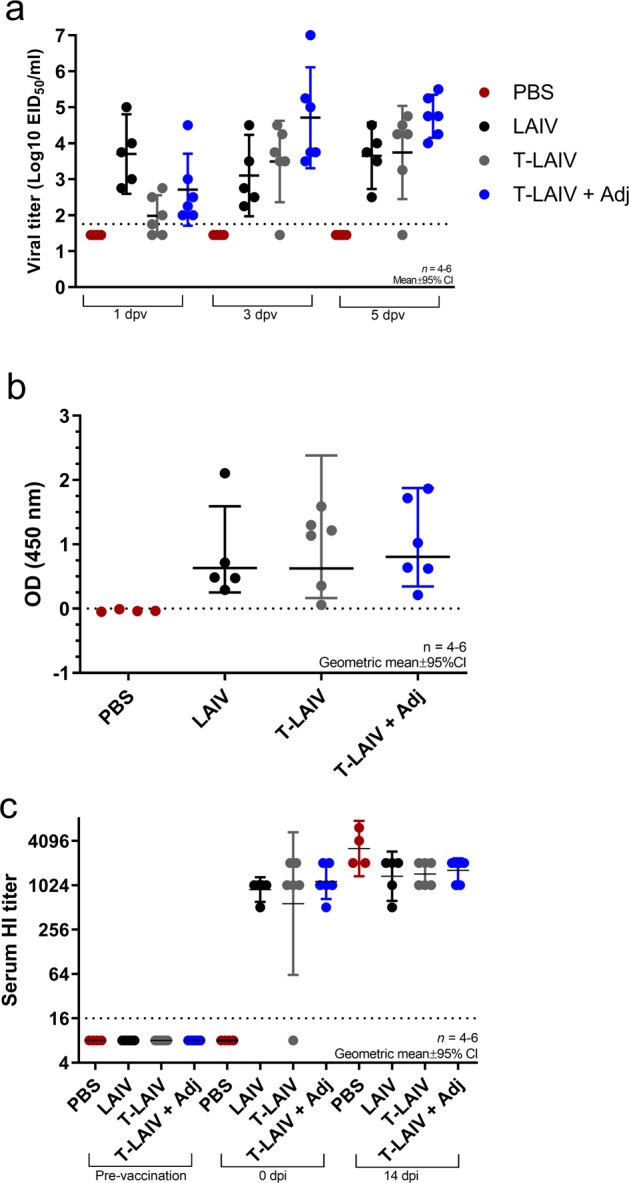
Infectivity and immunogenicity of T-LAIV in ferrets. **a** Vaccine virus detected in ferret nasal washes post-vaccination. Virus titer was determined by titration in embryonated chicken eggs. Kruskal–Wallis test with a post hoc Dunn’s multiple comparisons test. Error bars indicate mean ± 95% CI. *n* = 4–6. **b** Mucosal antibody response detected in ferret nasal washes post-vaccination. Nasal mucosa immunoglobulin response detected in ferret nasal washes by ELISA (32 dpv). Kruskal–Wallis test with post hoc Dunn’s multiple comparisons test. Error bars indicate geometric mean ± 95% CI. *n* = 4–6. **c** Seroconversion of ferrets following vaccination with LAIV, thermostable LAIV (T-LAIV), T-LAIV + adjuvant, or PBS. Serum hemagglutinin antibody titers pre-vaccination, post-vaccination, and post-challenge were determined by hemagglutinin-inhibition assay. Error bars indicate geometric mean ± 95% CI. *n* = 4–6.

Following verification of the stability and in ovo infectivity of PBV-stabilized T-LAIV, we assessed the replication ability of dry-powder T-LAIV in the upper respiratory tract of ferrets. Naive ferrets were intranasally vaccinated with 1 × 10^7.0^ EID_50_ liquid LAIV, T-LAIV, T-LAIV + Advax™ adjuvant (T-LAIV + Adj), or sham vaccinated with phosphate-buffered saline (PBS), and nasal washes were collected 1, 3, and 5 days post-vaccination (dpv). T-LAIV was delivered as a dry powder blown into the nares of anesthetized ferrets using an inhaler device, whereas liquid LAIV or PBS was delivered by droplet into the nares of anesthetized ferrets. Liquid LAIV efficiently replicated in the upper respiratory tract 1 dpv (mean: 10^3.6^ EID_50_/ml), with similar titers of virus detected in nasal washes 3 and 5 dpv (mean: 10^3.2^ and 10^3.8^ EID_50_/ml, respectively) (Fig. 2a). Compared to liquid LAIV, reduced vaccine virus titers were detected at 1 dpv in ferrets administered T-LAIV and, to a lesser extent, T-LAIV + Adj (mean: 10^2.0^ and 10^2.7^ EID_50_/ml, respectively). However, this apparent lag in virus replication disappeared by 3 dpv and the highest vaccine virus titers at days 3 and 5 were observed in the T-LAIV + Adj group. T-LAIV + Adj and T-LAIV mean virus titers detected at days 3 (10^4.7^ and 10^3.5^ EID_50_/ml, respectively) and 5 post-vaccination (10^4.8^ and 10^3.7^ EID_50_/ml, respectively) were higher or equivalent to virus titers in nasal washes of liquid LAIV-inoculated ferrets. Detection of high virus titers when using T-LAIV demonstrates feasibility of dry-powder LAIV delivery and suggests that the dry-powder T-LAIV, when formulated with or without Advax™ adjuvant, is likely to induce a robust immunogenic response comparable to or exceeding that of liquid LAIV.

### Vaccination with T-LAIV elicits robust mucosal and serum antibody responses

To assess the immunogenicity of T-LAIV, mucosal antibody secretion and serum HI antibody titers were determined. Vaccination of ferrets with T-LAIV (A/17/Texas/2012/30 (H3N2)) with and without Advax™ adjuvant induced significant mucosal IgG responses compared to PBS vehicle, with nasal wash IgG levels comparable to those induced by liquid LAIV (Fig. 2b). Similarly, vaccination resulted in robust induction of protective levels of serum HI antibodies in all vaccination groups (HI titer geometric means: liquid LAIV, 912; T-LAIV, 574; T-LAIV + Adj, 1149) (Fig. 2c). One ferret failed to seroconvert in the T-LAIV vaccination group; however, no infectious T-LAIV virus was detected in the nasal washes of this ferret, suggesting a technical vaccine delivery failure, which is understandable given the difficulty in blowing powder into the small nares of ferrets. These results demonstrate that T-LAIV was immunogenic when formulated with or without Advax™ and elicited robust mucosal IgG and serum HI responses.

### T-LAIV vaccination is efficacious against influenza A virus challenge

**Fig. 3:**
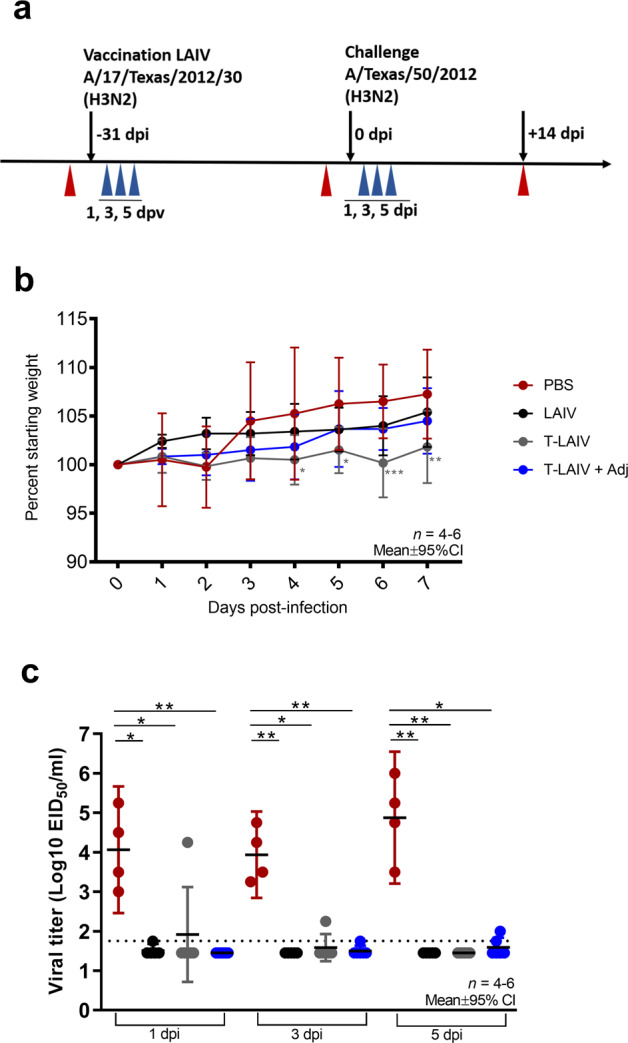
Efficacy of T-LAIV following challenge with A/Texas/50/2012 (H3N2). **a** Study design. Four to six 12-month-old ferrets were intranasally vaccinated with 1 × 10^7^ EID_50_ liquid LAIV, T-LAIV, T-LAIV + Advax adjuvant, or PBS. Ferrets were challenged with antigenically matched influenza virus (A/Texas/50/2012 (H3N2)) 4 weeks post-vaccination. Nasal washes were collected post-vaccination and post-challenge (blue arrows). Whole blood was collected pre-vaccination, post-vaccination/pre-challenge, and post-challenge (red arrows). **b** Weight change (percent) post-challenge with A/Texas/50/2012 (H3N2). Immunized ferrets were challenged intranasally with 1 × 10^7^ PFU A/Texas/50/2012 (H3N2) and weights were recorded daily for 7 days post-inoculation. Percent weight change was compared to PBS challenge group. Two-way ANOVA with Dunnett’s multiple comparisons test. Error bars represent mean ± 95% CI. *p < 0.05. *n* = 4–6. **c** Viral loads detected in ferret nasal washes post-challenge with A/Texas/50/2012 (H3N2). Virus titer was determined by titration in embryonated chicken eggs. Kruskal–Wallis test with a post hoc Dunn’s multiple comparisons test. *p < 0.05, **p < 0.01. Error bars indicate mean ± 95% CI. *n* = 4–6.

To determine the protective efficacy of thermostabilized LAIV against influenza A virus, ferrets were challenged by intranasal inoculation with 1 × 10^7^ EID_50_ of antigenically matched wild-type strain A/Texas/50/2012 (H3N2). Weight loss was monitored daily for 7 days and nasal wash virus titers were determined 1, 3, and 5 days post-inoculation (dpi) (Fig. 3a). Following challenge with A/Texas/50/2012 (H3N2), all vaccine groups maintained percent starting weight; however, compared to the PBS group, lower weights were detected for T-LAIV at 4–7 dpi (Fig. 3b). Post-challenge serum HI titers of all immunization groups further increased over pre-challenge levels (Fig. 2c), most notably in the PBS immunized group, which developed the highest HI titers post-challenge, consistent with a lack of protection in this group resulting in higher levels of influenza infection and, thereby, greatest immunologic stimulus.

To assess vaccine efficacy, virus titers in nasal washes were determined 1, 3, and 5 dpi. Virus shedding from the upper respiratory tract was detected in all ferrets in the unvaccinated (PBS) group, with an average of 10^4.1^, 10^3.9^, and 10^4.9^ EID_50_/ml of virus detected in nasal washes at days 1, 3, and 5 post-inoculation, respectively (Fig. 3c). With the exception of one ferret in the T-LAIV group that did not seroconvert (Fig. 2c), all vaccinated ferrets were protected following homologous challenge with the antigenically matched influenza strain (Fig. 3c). Interestingly, virus replication in the upper respiratory tract of the ferret that failed to seroconvert was reduced by 3 dpi and was not detected at 5 dpi, suggesting that mucosal immunity may have contributed to the control of viral infection in this animal, in the absence of detectable serum HI titer. In the seropositive vaccinated ferrets, viral shedding from the upper respiratory tract was significantly reduced to levels at or below the limit of detection, resulting in up to a 16 log2-fold reduction in virus shedding for LAIV, T-LAIV, and T-LAIV + Advax (1 dpi, 4–14 log2-fold reduction; 3 dpi, 9–12 log2-fold reduction; 5 dpi, 16 log2-fold reduction, respectively, vs. the PBS group). There was no significant difference in virus shedding between liquid LAIV, T-LAIV, or T-LAIV + Advax, demonstrating that PBV thermostabilization of LAIV was not detrimental to vaccine immunogenicity or protective efficacy in ferrets.

## Discussion

In this study, we assessed the immunogenicity and efficacy of a PBV-generated, dry-powder thermostable delta inulin (Advax™)-adjuvanted LAIV in the ferret model. Collectively, we demonstrated that PBV-generated, dry-powder T-LAIV was thermostable when stored at an ambient temperature (25 °C), induced robust immunological responses in ferrets, in particular when formulated with Advax™ adjuvant, and was efficacious against homologous challenge with an antigenically matched strain. T-LAIV infectivity was not adversely affected by the PBV stabilization process, in vitro or in vivo, nor by formulation with Advax™ adjuvant. Moreover, T-LAIV efficiently replicated in the upper respiratory tract of ferrets following dry-powder intranasal delivery, which resulted in protective immunity from (homologous) challenge with A/Texas/50/2012 (H3N2).

The World Health Organization categorizes vaccines from A to E, according to their relative heat sensitivity^48^. Influenza vaccines are highly sensitive to heat (group B classification), and any interruption to the vaccine supply cold chain is likely to compromise the potency and efficacy of the vaccine. Further, cold-chain requirements of the leading COVID-19 pandemic vaccine candidates (Moderna −20 °C; Biontech/Pfizer −80 °C) and their short shelf life at ambient temperature have been identified as challenges for their distribution and storage^49^. Thermostable dry-powder formulations could alleviate the need for maintenance of vaccines in a continuous cold chain throughout the delivery process. Enabling vaccine to remain safe and effective without refrigeration at delivery points can make the vaccine supply chain more efficient and effective at reaching everyone who needs immunization^50^.

Vaccines are commonly stabilized by spray or freeze-drying in the presence of stabilizing protectors, such as carbohydrates^26,51^. Recently, stabilization of herpes simplex virus 2 and influenza A virus vaccines was achieved by simple evaporative drying (also known as desorption) in the presence of carbohydrates^52^. Unfortunately, desorption is a diffusion-limited process that is difficult to scale up for industrial purposes. Thus, there is a need for alternative scalable drying technologies, such as PBV technology, which allow high-volume production of thermostable vaccines and other fragile biopharmaceuticals.

Several PBV-generated dry-powder, thermostable live attenuated vaccines have been developed, including measles, rubella, yellow fever (unpublished), and rabies^53,54^. Dry-powder vaccines developed by PBV have been demonstrated to be stable at ambient temperature for at least 23 months^53^. In this study, the PBV-generated T-LAIV was highly stable when stored at an ambient temperature (25 °C) for at least 1 year, exhibiting minimal loss of titer. When stored at an elevated temperature (37 °C) for up to 1 year, T-LAIV lost ~1.5 logs of viral infectivity. The demonstrated thermostability of PBV-generated T-LAIV could allow vaccine shipping without refrigeration and storage at the point of delivery for months under controlled temperature chain conditions. As controlled temperature chain conditions do not require refrigeration, PBV-generated vaccines could be distributed, stored, and delivered in areas without reliable refrigeration^50^. For pandemic preparedness, LAIV vaccines with the level of thermostability demonstrated in this study could potentially be kept long term in a pandemic stockpile under refrigerated conditions and released for shipping, local storage, and delivery over several months without refrigeration.

Naturally acquired influenza infection of humans occurs predominately by infection of the mucosal surface of the upper respiratory tract. As LAIV mimics the natural route of influenza infection, it exhibits several advantages compared to injected IIVs, including localized immune responses at the site of infection resulting in robust mucosal antibody response^11^ and cell-mediated immune response even in the absence of detectable serum antibodies^12^. Choice of LAIV MDV backbone may influence immunological outcomes following vaccination. Multiple studies have directly compared the US and Russian LAIV backbones; comparisons of the replication ability of *ca* A/Ann Arbor/6/60, *wt* A/Leningrad/134/57, *ca* A/Leningrad/134/17/57, and *ca* A/Leningrad/134/47/57 in human bronchial epithelial cells revealed that the Russian MDVs replicated to significantly higher titers than A/Ann Arbor/6/60^55^. Reduced replication ability of A/Ann Arbor/6/60 in human nasal epithelial cells has been attributed to the presence of an A86S mutation in the matrix protein (M2)^56^. High vaccine virus titers are beneficial to inducing robust immunity—particularly in the elderly and in pre-immune populations that likely harbor cross-reactive antibodies. In addition, immunogenicity studies in mice revealed that A/Leningrad/134/17/57 elicits a superior immunological response as compared to A/Leningrad/134/47/57 and A/Ann Arbor/6/60^57^, further supporting that the Russian Leningrad MDV backbone used herein may be favorable over A/Ann Arbor/6/60. Finally, reversion of an Ann Arbor-backbone LAIV (FluMist™) to a phenotype capable of inducing disease in mice following serial passage of the vaccine virus at increasing temperatures^58^ highlights the need to consider alternative LAIV backbones for future LAIV formulations. Indeed, a Leningrad-based LAIV developed by the Institute of Experimental Medicine (Russia) and subsequently sublicensed via a World Health Organization influenza technology transfer initiative^59^ has been demonstrated to be safe and well tolerated in human clinical trials^59,60^.

Traditionally, a correlate of protection against antigenically related influenza strains is an HI titer > 40 or a fourfold increase in serum HI antibody response following vaccination with inactivated trivalent/quadrivalent influenza vaccine formulations^61^. However, LAIV has been shown to be protective in the absence of serum HI antibody response. Herein, vaccination of ferrets with a Leningrad-backbone liquid LAIV or PBV-generated T-LAIV (stored at room temperature for ~3 months) was shown to induce detectable mucosal IgG and serum HI antibody responses. Intranasal vaccination of humans with influenza vaccines has been shown to induce both IgA- and IgG-neutralizing antibodies in nasal washes^62^. Vaccination of ferrets with T-LAIV elicited mucosal IgG antibody response at levels similar to liquid LAIV, suggesting that PBV thermostabilization of LAIV does not impair mucosal immunity induced in the host. Moreover, vaccination of ferrets with T-LAIV induced protective serum HI antibody response, similar to that induced by liquid LAIV. One ferret in the T-LAIV group did not seroconvert, potentially due to a reduced dose related to the difficulty of administering nasal dry powder to ferrets. A mucosal antibody response was detected in this ferret, albeit at a low level, and virus shedding on 3 and 5 dpi was markedly curtailed. Mucosal immunity may have played a role in effectively reducing the viral burden and duration of infection in the upper respiratory tract of this ferret. Although it is anticipated that the immunogenicity of T-LAIV stored at room temperature for 1 year would be similar to that stored for 3 months, as used in this study, this warrants further investigation. Also assessing the durability of the immune response following vaccination with T-LAIV would be of interest.

The inclusion of adjuvants with vaccines is a means to increase breadth of vaccine effectiveness and increase efficacy in at-risk populations (e.g., elderly populations), as well as to enable dose sparing^63^. Mucosal adjuvants have added benefits of improving mucosal antibody responses, which are important in preventing viral respiratory infection and have been shown to improve the breadth of protective immunity^64^. Here we assessed the impact of co-formulation of Advax™, a polysaccharide adjuvant derived from delta inulin, with T-LAIV on immune responses and vaccine efficacy in ferrets. Advax™ has been shown to increase influenza-specific immune responses and protection, as well as enable dose sparing in murine studies^42,65^. A recent Phase I trial of trivalent IIV formulated with Advax™ demonstrated both safety and potential dose sparing^47^. When co-formulated with T-LAIV as a mucosal dry-powder vaccine, Advax™ appeared to enhance nasal T-LAIV titers over T-LAIV alone, an effect that warrants further investigation. Although the ability of Advax™ to provide T-LAIV dose sparing was not studied here, this could also be the subject of future studies, given the trends observed. Advax™ in other studies has been shown to induce a strong T-cell response^39,66^ and to enhance heterologous virus protection^67,68^, so it would be interesting to assess whether Advax™ can similarly enhance the ability of T-LAIV to provide protection against a heterologous influenza virus challenge in future studies.

Vaccination by liquid nasal spray can result in suboptimal vaccine deposition. The large droplets administered by nasal spray devices tend to accumulate in the nares and much of the dose is often wasted by dripping out of the nose^69^. Also, liquid nasal sprays may require an experienced vaccinator, as differences in the force and speed of plunger depression results in variable droplet size^70^, and angular placement of the spray tip can significantly affect vaccine deposition^71^. Studies in anatomic models of nasal airways have shown dry-powder nasal delivery markedly improves vaccine deposition compared to liquid nasal spray delivery^72^. Although there are not currently any commercial dry-powder intranasal vaccines, the ease of administration, transport, and storage of PBV-generated dry-powdered vaccines could make these formulations an attractive alternative to liquid formats. Moreover, the ability of the patient to self-administer T-LAIV powder via the inhaler device tested in this study increases convenience and eliminates parenteral injection, which may lead to an increased vaccine uptake.

Collectively, PBV-generated, dry-powder T-LAIV was immunogenic and was efficacious against homologous influenza virus challenge with an antigenically matched strain. These studies provide a proof-of-concept for the development of thermostable dry-powder vaccine countermeasures against influenza virus. The addition of Advax™ enhanced the effectiveness of T-LAIV; potential benefits that could be explored in the future are dose sparing and/or heterologous challenge studies. Hence, T-LAIV formulated with delta inulin adjuvant represents a promising human influenza vaccine candidate for further development. Finally, although animal studies relied on controlled delivery of the dry-powder vaccine, the blister-pack and dry-powder delivery device used for these studies are designed for self-delivery, presenting the opportunity for self-administration of stockpiled PBV-stabilized LAIV in the case of an influenza pandemic.

## Methods

### Ethics statement

All animal experiments were reviewed and approved by the University of Georgia Institutional Animal Care and Use Committee and complied with the National Institutes of Health guide for the care and use of Laboratory animals.

### Viruses and cells

Seasonal influenza A virus, A/Texas/50/2012 (H3N2), and an antigenically matched LAIV virus A/17/Texas/2012/30 (H3N2) were used in this study. The LAIV virus contained the hemagglutination (HA) and neuraminidase from A/Texas/50/2012 on the A/Leningrad/134/17/57 (H2N2) backbone. Virus stocks were generated in E9-11 ECEs, allantoic fluid collected, clarified by centrifugation, and stored at −80 °C. Virus titers were determined by titration in 9–11-day-old ECEs or Madin–Darby canine kidney cells (ATCC CCL-34) in MEM (Corning, Cellgro, New York, USA) supplemented with 10% fetal bovine serum (Atlanta Biologicals; Georgia, USA), 1× antibiotic–antimycotic (ThermoFisher Scientific, Gibco, Massachusetts, USA) and 2 µg/ml TPCK-treated trypsin (Worthington Biochemicals, New Jersey, USA).

### Thermostabilization of LAIV using PBV

Liquid LAIV was stabilized by PBV, a foam-drying process that simultaneously sublimes, boils, and evaporates liquid samples at ≥ −10 °C and ≤3 Torr^28,53^, to efficiently remove liquid and create a thermostable biologic. Liquid LAIV was combined 1 : 1 with a protective carbohydrate solution (30% sucrose and 10% mannitol) and underwent primary drying by boiling under vacuum at near-subzero temperatures, prior to secondary stability drying at elevated temperatures. The secondary stability drying phase of LAIV included 24 h at 45 °C to achieve glass transition phase; the glass state was achieved by rapidly cooling the dried LAIV formulation below 37 °C. In the glass state, molecular mobility is essentially arrested^29^, preventing degradation of immobilized LAIV during storage and conferring long-term stability. The dry-powder T-LAIV was micronized, then sieved to 20–60 µM mesh size, and portions were aliquoted for stability testing. Finally, dry-powder T-LAIV was mixed with sieved (>60 µM) Lactohale lactose carrier (DFE Pharma, Goch, Germany) at a dose of 1 × 10^7^ EID_50_ and aliquoted into blister packs with or without dried Advax™ adjuvant^73^.

### T-LAIV infectivity assay

T-LAIV was incubated at 25 °C or 37 °C and liquid LAIV was stored at −80 °C for up to 52 weeks (*n* = 3). T-LAIV was reconstituted with 1 ml PBS and titrated tenfold in E9-11 ECEs. Briefly, ECEs were inoculated with 200 µl of reconstituted virus (five eggs per dilution). Eggs were incubated at 32 °C with humidity for 3 days. ECEs were chilled overnight at 4 °C and HA assay performed on allantoic fluid. EID_50_ was calculated according to Reed–Muench method.

### Animals and study design

Groups of four to six 12-month-old male ferrets (Triple F Farms, Pennsylvania, USA) were vaccinated by intranasal administration of 1 × 10^7^ EID_50_ liquid LAIV (LAIV) (*n* = 5) (1 ml), T-LAIV powder (*n* = 6), or T-LAIV + Adj powder (*n* = 6). The T-LAIV lots used were stored for 3 months at room temperature prior to vaccination. One group of ferrets remained unvaccinated (PBS) (*n* = 4) (1 ml). T-LAIV and T-LAIV + Adj was delivered as a dry powder blown into the nares of anesthetized ferrets using an inhaler device fitted with a rubber bulb (Creare LLC, New Hampshire, USA) (inhaler device described in detail in US Patent No. 10,099,024^74^) The inhaler device enables administration of dry-powder T-LAIV to the respiratory tract without the need for reconstitution. Liquid LAIV or PBS was delivered by droplet into the nares of anesthetized ferrets.

Nasal washes were collected at 1, 3, and 5 dpv and stored at −80 °C. Four weeks later, ferrets were challenged intranasally with 1 × 10^7^ PFU of antigenically matched A/Texas/50/2012 (H3N2). Nasal washes were collected at 1, 3, and 5 dpi and stored at −80 °C. Whole blood was collected pre-vaccination, pre-challenge, and post-challenge, and serum separated for serological analysis.

### Immunoglobulin ELISA

Post-vaccination nasal mucosal IgG response (32 dpv/1 dpi) to antigenically homologous virus strain was assessed by enzyme-linked immunosorbent assay (ELISA). ELISA plates were coated with 1 : 100 A/Texas/50/2012 and incubated overnight at 4 °C, washed three times with PBS, and blocked with 1% bovine serum albumin in PBS. Nasal wash samples were diluted fourfold (in duplicate) and incubated overnight at 4 °C. Plates were washed three times with PBST and incubated with 0.2 µg/ml biotinylated goat anti-ferret IgG (γ-chain specific) (Sigma Aldrich, Missouri, USA), followed by 0.4 µg/ml Streptavidin-HRP (Vector Laboratories, California, USA). Nasal mucosal anti-A/Texas/50/2012 immunoglobulin was detected using TMB substrate (Vector Laboratories). Results were read on an Epoch microplate spectrophotometer at 450 nm.

### Serology

Ferret sera were heat inactivated, treated with receptor-destroying enzyme (Denka Seiken, Osaka, Japan), and absorbed with 10% turkey erythrocytes. HI assay was performed according to OIE guidelines^75^ using turkey erythrocytes. The reciprocal of the highest serum dilution resulting in complete inhibition of agglutination was considered the HI titer.

### Virology

Nasal washes were titrated tenfold in E9-11 ECEs. Briefly, ECEs were inoculated in quadruplicate with 100 µl of titrated nasal wash fluid. Eggs were incubated at 32 °C (LAIV virus) or 37 °C (A/Texas/50/2012 (H3N2)) with humidity for 3 days. ECEs were chilled overnight at 4 °C and HA assay performed on allantoic fluid.

## Statistical analyses

Statistical analyses were performed using GraphPad Prism, version 9.0.0 (GraphPad Software, Inc., USA). Data from T-LAIV infectivity assay and percent weight were analyzed using a two-way analysis of variance. Viral titers, serum HI titers, and mucosal IgG responses were analyzed using a Kruskal–Wallis test with a post hoc Dunn’s multiple comparisons test.

### Reporting summary

Further information on research design is available in the Nature Research Reporting Summary linked to this article.

## Supplementary information

Reporting Summary

## Data Availability

The data that support the findings of this study are available from the corresponding author upon reasonable request.
